# Spatial mapping of human colonic niches reveals rapid, mucus-specific microbiota disruption after bowel cleansing

**DOI:** 10.1080/19490976.2026.2635866

**Published:** 2026-02-25

**Authors:** Bahtiyar Yilmaz, Sarah Moulin, Benjamin Heimgartner, Hai Li, Markus Geuking, Pascal Juillerat, Benjamin Misselwitz, Andrew J. Macpherson, Reiner Wiest

**Affiliations:** a Department of Visceral Surgery and Medicine, Inselspital, Bern University Hospital, University of Bern, Bern, Switzerland; b Department for Biomedical Research, University of Bern, Bern, Switzerland; c Bern Center for Precision Medicine (BCPM), University of Bern, Bern, Switzerland; d Center for Advanced Interdisciplinary Science and Biomedicine of IHM, School of Basic Medical Sciences, Division of Life Sciences and Medicine, University of Science and Technology of China, Hefei, People's Republic of China; e Department of Microbiology, Immunology and Infectious Diseases, Cumming School of Medicine, University of Calgary, Calgary, Alberta, Canada; f Medical Department II, University Hospital, LMU, Munich, Germany

**Keywords:** Intestinal microbiota, bowel cleansing, purging effect, mucus suction

## Abstract

Bowel preparation is routinely performed before colonoscopy, yet its immediate effects on the spatial organization of the colonic microbiota at the mucosal interface remain poorly resolved. Here, we introduce a high-resolution endoscopic mucus-harvesting approach, combined with luminal aspirates and mucosal biopsies, to generate a high-resolution, within-subject trajectory of microbiota alterations across distinct colonic niches in healthy adults over the first 24 hours after purging. While luminal bacterial communities remained remarkably stable, with no significant changes in alpha or beta diversity and proportional washout of taxa. In contrast, mucus-associated and mucosal communities underwent a rapid but reversible ecological restructuring, characterized by immediate post-cleansing shifts in composition and transient blooms of Proteobacteria, particularly *Enterobacteriaceae*. These perturbations were strongest in the 0−12-hour window and varied by individual, consistent with the dominance of personalized baseline microbial signatures. Critically, spatially resolved sampling revealed a key refinement: the *Enterobacteriaceae* expansion was confined almost exclusively to the superficial mucus layer, a glycan-rich, dynamically oxygenated compartment that is particularly susceptible to mechanical disturbance during lavage, whereas deeper mucus and mucosa-associated communities remained comparatively stable. By 24 hours, both mucosal and mucus-associated microbiota had largely returned to their individualized pre-cleansing configurations, indicating rapid ecosystem resilience and suggesting that the deeper mucus layer functions as a protected microbial reservoir that reseeds the epithelium and lumen once normal physiology is restored. This compartment-specific recovery trajectory contrasts with the prolonged dysbiosis typically observed after antibiotics or infection, underscoring the need for spatially precise sampling to interpret microbiome data collected during clinical endoscopy. Together, these findings establish an endoscopic strategy for probing microbe-mucus interactions in humans and provide a conceptual and methodological framework for interpreting microbiome data obtained during clinical endoscopy.

## Introduction

Maintaining a stable relationship between the intestinal microbiota and the host is essential for gut homeostasis and systemic health. Disruption of this equilibrium can promote intestinal inflammation, metabolic imbalance, or systemic pathology.^1-6^ Much of this interaction is mediated through the spatial organization of the microbiota along the gut axis, which remains difficult to resolve in humans due to technical constraints on sampling. Luminal communities differ substantially from the consortia embedded within the mucus and mucosal layers,^7^^,^^8^ which constitute autochthonous microbial niches with distinct metabolic profiles, replication dynamics, and host interactions.^9-12^ Experimental work has shown that identical bacterial species elicit compartment-specific transcriptional and physiological responses depending on whether they colonize the lumen or the mucus,^10^ underscoring that intestinal microbiota cannot be viewed as a uniform entity.

Despite the biological importance of these niches, access to spatially resolved microbial communities in humans remains technically challenging. Endoscopic biopsies, the dominant method to study mucosa-associated microbiota, mix bacteria from loosely adherent mucus, the outer mucus layer, and the deeper mucosa.^13-15^ High host DNA content can further obscure microbial profiles,^16^ and the term “mucosa-associated microbiota” therefore often refers to a heterogeneous mixture of microbial strata whose relative contributions remain unknown. Dedicated mucus sampling has rarely been attempted in vivo, leaving the microbial architecture of the mucus layer (in particular its outer, dynamic fraction) largely unexplored. This gap reflects not only conceptual neglect but also the absence of endoscopic strategies capable of selectively sampling the mucus compartment in humans.

Bowel preparation introduces an additional layer of complexity. Oral purgatives accelerate intestinal transit, alter nutrient availability, and physically wash out luminal biomass. These preparations are indispensable for colonoscopy, yet they are also widely used, implicitly or explicitly, in microbiome research. A large proportion of human microbiota studies rely on biopsy samples collected after bowel cleansing,^7^^,^^8^ making it critical to understand how purging alters the communities that researchers seek to measure. The perturbation is not purely luminal: bowel preparation affects the physicochemical environment at the mucosal interface, where changes in oxygen tension, mucus structure, and microbial load may differentially impact the adherent microbiota.

Several studies have examined the effects of bowel cleansing on the gut microbiome, but the findings are inconsistent and most work has focused on fecal or luminal samples.^17-20^ Only a few studies have evaluated mucosa-associated communities using biopsies,^19^^,^^21^ and these investigations were constrained by heterogeneity in timing, post-prep sampling intervals, and cleansing regimens. Reported responses range from minimal diversity changes to substantial community shifts, with transient increases in Proteobacteria or Bacteroidetes and decreases in Firmicutes, Actinobacteria, and Tenericutes.^22-27^ Importantly, none of these studies examined the short-term kinetics of recovery across distinct colonic compartments, nor did any explicitly target the mucus layer as a discrete ecological niche. Given that the mucus is believed to both shelter autochthonous microbial communities and buffer environmental perturbations, the absence of mucus-specific data limits our understanding of microbiome resilience during bowel preparation.

To address these gaps, we developed and applied a targeted endoscopic mucus-harvesting strategy to conduct a multi-compartment analysis of the human colonic microbiota before and after bowel cleansing. This approach isolates the outer mucus layer and enables spatial deconvolution of luminal, mucus-associated, and mucosa-associated niches. By integrating mucosal biopsies, luminal content, and mucus aspirates collected at serial time points over 24 hours, we sought to define the immediate impact of purging on niche-specific microbial communities and to characterize their short-term recovery dynamics. This approach provides a more refined understanding of microbiota resilience at the mucosal interface and clarifies how bowel preparation alters the microbial environments routinely sampled in clinical and research settings.

## Methods

### Study design and participants

This study involved five healthy adult volunteers (four males, one female; age range 40−60 years), none of whom had taken antibiotics or chronic medications for at least six months prior to enrollment. One participant underwent the protocol twice over a one-year interval. All procedures were approved by the Bern Cantonal Ethics Commission (KEK 336/2014), and all participants provided written informed consent.

**Figure 1.**
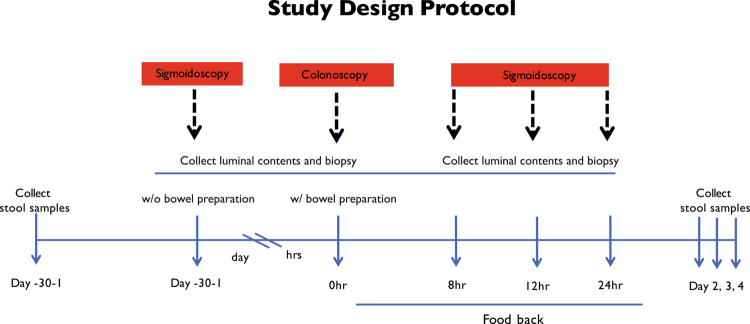
Study protocol. Sigmoidoscopy and colonoscopy procedures were used to collect samples before bowel cleansing (unpurged) and after bowel cleansing (purged). At baseline, only stool samples were collected. Following bowel preparation, luminal content and biopsy samples were obtained at defined time points, as illustrated in the schematic.

To characterize the short-term impact of bowel cleansing on luminal and mucosa-associated microbiota, we implemented a longitudinal sampling design comprising pre-cleansing (“unpurged”) sigmoidoscopy and four post-cleansing (“purged”) time points: immediately after colonoscopy (0 h), and at 8 h, 12 h, and 24 h thereafter (Figure 1). Samples included luminal content, mucosal biopsies, and mucus aspirates.

A separate cohort of 21 individuals undergoing diagnostic sigmoidoscopy was used to develop and validate the mucus-harvesting method under both unpurged and purged conditions.

### Bowel cleansing protocol

Bowel preparation was performed using PicoPrep® (10 mg sodium picosulfate, 3.5 g magnesium oxide, 12 g citric acid per sachet) administered as a split-dose regimen, as previously described.^28^ Upon dissolution, magnesium citrate acts as an osmotic agent, drawing water into the intestinal lumen, whereas sodium picosulfate increases colonic peristalsis. This preparation typically induces 10−20 diarrheal evacuations and constitutes a marked perturbation of the colonic environment. On the day of purging, participants were permitted up to four 240 mL servings of clear liquids before endoscopy. A regular diet was resumed immediately following recovery from colonoscopy.

### Endoscopic procedures and multi-compartment sampling

Subjects underwent several sigmoidoscopy procedures using an Olympus EVIS EXERA III (CF-HQ190L/I) video colonoscope with an advanced Dual Focus optical system. The simplified protocol is depicted in Figure 1.

Pre-cleansing recto-sigmoidoscopy was performed without sedation within 30 days prior to bowel preparation. Luminal content (=consistent fecal material) and mucosal biopsies were collected from the rectosigmoid region using standard endoscopic forceps (using different forceps for each site, respectively). Samples were transferred immediately into RNAlater and stored at –80 °C until further processing.

Following completion of bowel preparation, participants underwent colonoscopy under conscious sedation with propofol (Disoprivan® 1%). Samples were obtained from the ileum, caecum, transverse colon, descending colon, and rectum. Additional recto-sigmoidoscopies were performed at 8 h, 12 h, and 24 h after the procedure to collect luminal content and mucosal biopsies from identical anatomic regions. In some cases, luminal content was difficult to visually identify in the rectosigmoid region. Under these circumstances, standard endoscopic biopsy forceps were used to sample luminal material by targeting visibly darker, brownish fluid accumulations (“lakes”) at sites with maximal discoloration, indicative of residual fecal material. The forceps were gently closed within these regions to collect luminal contents.

All material was preserved in RNAlater and stored at –80 °C.

### Analytical scope and anatomical stratification

Although samples were collected from multiple gastrointestinal locations during colonoscopy (including ileum, caecum, transverse colon, descending colon, and rectum), the primary longitudinal and recovery analyses were intentionally restricted to the rectosigmoid region, which was the only anatomical site sampled consistently across all pre- and post-cleansing time points. This restriction ensured within-subject comparability over time. Samples from more proximal colonic segments were used for descriptive and cross-sectional analyses where indicated, but were not included in temporal modeling of microbiota recovery.

### Mucus suction protocol for outer mucus layer harvesting

(Supplementary Video 1 in this link). To selectively sample the outer mucus layer, we developed an endoscopy-guided aspiration technique. A 6-mm tracheobronchial aspiration catheter was modified by cutting its distal tip diagonally to maximize surface contact. Under endoscopic visualization, the catheter was introduced trans-anally and positioned tangentially against the rectal mucosa. A 50-mL syringe attached to the proximal end generated negative pressure, enabling aspiration of mucus directly from the mucosal surface. Under continuous endoscopic visualization, negative pressure was increased in a stepwise manner until visible movement of mucus into the catheter was observed. If initial aspiration yielded insufficient material, the catheter was gently repositioned along the mucosal surface while maintaining constant negative pressure to enhance mucus recovery.

The mucus-harvesting protocol was implemented using a standardized, stepwise procedure designed to enable stratified sampling of the rectal mucosal interface. First, a baseline mucosal biopsy (Biopsy 1) was obtained prior to any manipulation of the mucus layer. In a predefined subgroup, the superficial outer mucus layer was then mechanically disrupted by applying a focused 20-second aqua-jet flush through the endoscopic working channel. During this step, the endoscope tip was maintained at a fixed position, and irrigation was delivered using an AquaJet pump (Duomed Swiss AG) set to ≥50% of maximal output.

Immediately thereafter, targeted mucus aspiration was performed at the same anatomical site without repositioning the endoscope. A modified aspiration catheter was placed in direct contact with the mucosal surface, and negative pressure was applied to selectively remove residual mucus. Following catheter disengagement, a localized haemorrhagic point was consistently observed, indicating the area subjected to maximal suction force. A second biopsy (Biopsy 2) was subsequently obtained from this exact site to sample the deeper mucosa-associated microbial community exposed after mucus removal.

In an additional predefined subgroup, a second sequential mucus aspiration was performed at the identical location to assess technical reproducibility and to quantify depletion of superficial mucus-associated microbial communities.

### DNA extraction

DNA extraction from stool/lumen content, and biopsy samples were performed using QIAamp DNA stool kit (Qiagen) and AllPrep DNA/RNA Mini Kit (Qiagen), respectively. Briefly, human fecal samples are collected into 2 ml microfuge tubes and stored at –20 °C prior to DNA extraction protocol. Total DNA is isolated from fecal samples using the QIAamp DNA Stool Kit (Qiagen) according to the manufacturer”s instructions, with additional modified steps. Briefly, 100 mg fecal pellets are homogenized in 500 μl Buffer ASL buffer by bead-beating step using Retsch Tissue Lyser for 3 min at 30 Hz and with two additional 95 °C lysis steps. Afterwards, samples are incubated with 200 μl Lysis Buffer for Gram+ (20mg/ml lysozyme Sigma 62970-5G-F); 20 mM Tris-HCl, pH 8.0; 2 mM EDTA; 1.2% Triton (Sigma-Aldrich) treatment. After adding 500 μL Buffer ASL, the manufacturer’s protocol was followed. Total DNA is eluted in 30 μl RNase-free water. Samples are stored at –20 °C until the amplicon PCR was performed. In contrast, biopsy samples were initially collected into 2 ml microfuge tubes containing RNAlater (Sigma-Aldrich) and stored at –80 °C prior to DNA extraction protocol that was carried out using AllPrep DNA/RNA Mini Kit (Qiagen) according to the manufacturer’s instructions.^29^ Briefly, 600 μL of Buffer RLT Plus *β*-mercaptoethanol with a metal bead was added to each tube containing the biopsy. Samples were then homogenized using the Retsch Tissue Lyser (Qiagen) at 30 Hz for 3 min, followed by 3 min of centrifugation at maximum speed (Eppendorf). Supernatants were transferred into all Prep DNA mini spin columns and centrifuged at 10.000 rpm for 30 sec. DNA attached to spin columns (Qiagen) was washed/desalted using 500 μL of Buffer AW1 and Buffer AW2. Then, DNA samples were eluted with 20−30 μl EB buffer (Qiagen) into 1.5 ml microfuge tubes. The concentrations and purity of the isolated DNA were assessed using a NanoDrop® (Thermo Scientific).

### Microbial community sequencing and analysis

The V5-V6 hypervariable region of the bacterial 16S rRNA gene was amplified using the KAPA HiFi HotStart ReadyMix DNA polymerase (Roche) from 200-1000 ng of template DNA, generating an expected amplicon of approximately 350 bp. Amplification was performed with barcoded, bacteria-specific primers. The forward primer carried a sample-specific barcode and the sequence 5’-CCATCTCATCCCTGCGTGTCTCCGACTCAGC-barcode-ATTAGATACCCYGGTAGTCC-3’, whereas the reverse primer was 5’-CCTCTCTATGGGCAGTCGGTGATACGAGCTGACGACARCCATG-3’.^30^

PCR conditions were as follows: initial denaturation at 94 °C for 5 min; 35 cycles of 94 °C for 1 min, 46 °C for 20 s, and 72 °C for 30 s; and a final extension at 72 °C for 7 min. Amplicons were visualized on a 1% agarose gel (2 h run) and purified using a QIAquick Gel Extraction Kit (Qiagen). Positive and negative controls were confirmed by gel electrophoresis but excluded from downstream sequencing. Amplicon concentrations were quantified with a Qubit 3.0 Fluorometer (Thermo Fisher Scientific), normalized to 26 pM, and pooled into a single library. Sequencing was performed on the Ion PGM™ System (Thermo Fisher Scientific) using the Ion PGM™ Sequencing 400 Kit and Ion 316™ v2 Chip.^31^

Raw sequence files were imported into the QIIME2 pipeline^32^ on the UBELIX high-performance computing cluster at the University of Bern, following established workflows.^33^ Read quality was inspected using *demux* summarize, which generates Q-score distributions based on random subsampling across base positions.

Within the DADA2 plugin, sequences were processed by removing primers, filtering low-quality reads, and eliminating chimeras, with quality evaluation based on Q-scores. Reads with Q > 30 were retained for downstream analysis. Although no external chimera-checking tool was applied, DADA2’s built-in consensus method^34^ was used to identify and remove chimeric sequences. After trimming, denoising, sequence correction, paired-end merging, and amplicon sequence variant (ASV) inference at 100% identity, we generated the table.qza (ASV count table) and rep-seqs.qza (ASV representative sequences).

Taxonomy assignment was performed using a pre-trained SILVA 132 classifier^35^ via the feature-classifier classify-sklearn method. Taxonomic composition and read distributions across ranks were visualized using taxa barplot and QIIME2 View (https://view.qiime2.org). Genus-level abundances were derived by collapsing the ASV table using taxa collapse, generating the *collapsed-table.qza* dataset.

Alpha diversity (species richness) was calculated using the Shannon and Simpson indices. Beta-diversity was assessed using Bray-Curtis dissimilarity at the genus level, based on relative abundances. Group differences in beta-diversity were evaluated using PERMANOVA with pairwise comparisons implemented via the *pairwiseAdonis* R package.^36^^,^^37^ To test for differences in dispersion among groups, we calculated multivariate homogeneity of group variances using *betadisper* from vegan package with PERMDISP2 as described by Anderson.^38^

For multivariable association testing, we applied MaAsLin2 with Benjamini–Hochberg false discovery rate correction.^39^^,^^40^ ASVs present in <10% of samples or with relative abundance <0.0001% were excluded to reduce sparsity. Associations were tested at the phylum, family, and genus levels using linear modeling on relative-abundance data. Subject identity was included as a random effect to account for repeated sampling, and covariates including age, sex, and time point were incorporated where applicable. In this context, analyses were performed at aggregated taxonomic levels (primarily genus level), enabling stable modeling of longitudinal associations across individuals while reducing sparsity. Following current best practices, rarefaction was not applied in downstream analyses.^41^

For pairwise, timepoint-specific differential abundance analyses, count-based modeling was performed using DESeq2.^42^ ASV-level feature tables generated after quality filtering were used as input. Prior to analysis, low-abundance features were filtered by retaining ASVs present in at least 30% of samples to reduce sparsity and improve statistical power. Raw count data were internally normalized in DESeq2 using a size factor estimated by the median-of-ratios method. Differential abundance testing was conducted using negative binomial generalized linear models, with time point specified as the main variable of interest. For each comparison, Wald tests were applied to estimate log2 fold changes and associated significance values. Multiple testing correction was performed using the Benjamini-Hochberg procedure to control the false discovery rate (FDR). ASVs with an adjusted q-value < 0.05 and an absolute log2 fold change >2 were considered significantly differentially abundant.

These analyses were used primarily to characterize acute, high-resolution ASV-level shifts between defined time points, whereas longitudinal and repeated-measures effects were modeled using MaAsLin2.

Taxonomic barplots, PCoA plots, and differential abundance outputs were generated using QIIME2, *phyloseq, ggplot2,* and QIIME2 View (https://view.qiime2.org/).

## Results

### The effect of bowel cleansing on altering the microbial composition

We first present luminal and conventional biopsy-based analyses as contextual reference compartments, before focusing on mucus-resolved microbial niches enabled by the high-resolution harvesting approach, which constitutes a central biological and methodological advance of this study. We analyzed 107 mucosal biopsy and luminal content samples from five volunteers, generating 4,126,271 high-quality 16S rRNA gene sequences after quality filtering (mean 38,563 reads per sample; range 1,161-190,722). These reads clustered into 79,752 ASVs, providing species-level resolution.^43^ Unless otherwise stated, longitudinal analyses focus on rectosigmoid samples, which were consistently available across all time points before and after bowel preparation.

**Figure 2.**
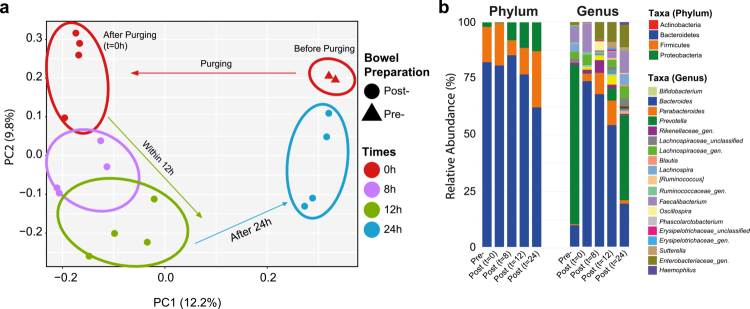
Temporal trajectory of microbiota changes from pre-cleansing to 24 hours post-cleansing in one volunteer. (a) Principal coordinate analysis (PCoA) illustrating descriptive, within-subject microbiota shifts from pre-cleansing to post-cleansing time points. Pre-cleansing samples consist of rectal and sigmoid colon biopsies obtained before bowel preparation (*n* = 2), whereas post-cleansing samples include luminal content and biopsies collected during colonoscopy (t = 0 h) and follow-up sigmoidoscopies at 8 h, 12 h, and 24 h (*n* = 4 per time point). This panel is shown to illustrate short-term microbiota dynamics and is not intended for statistical inference. (b) Relative abundance profiles at the phylum and genus levels for the same subject across all sampled time points.

A temporal trajectory from a representative participant showed that bowel cleansing induced a marked but short-lived perturbation in microbial composition. Beta diversity, as measured in biopsies and luminal contents, shows an immediate shift in community structure after purging in the sigmoid colon and rectum, followed by a progressive return toward the pre-cleansing configuration within 24 hours (Figure 2a). Taxonomically, this transient disturbance was characterized by a brief expansion of Proteobacteria, particularly *Enterobacteriaceae* and *Bacteroides*, and a corresponding reduction in *Prevotella* and members of *Ruminococcaceae* and *Lachnospiraceae* (Figure 2b). This outlines the impact of bowel cleansing on the microbiome and visualizes, on a case-by-case basis, the rapid kinetics of the microbial profile towards the restoration of normal consortia composition within one day after completing the purging.

**Figure 3.**
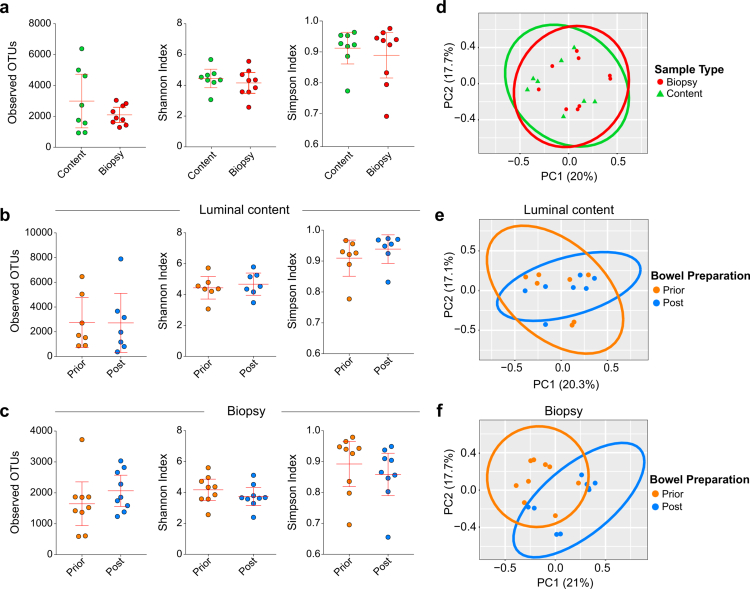
The effect of bowel cleansing on microbial diversity of biopsy and luminal content samples. Species richness in luminal content and biopsy samples collected prior to bowel cleansing was calculated using alpha diversity metrics (Observed ASVs, Shannon, and Simpson indices) in (a). Species richness was calculated for luminal content samples (*n* = 8) in (b) and for biopsies (*n* = 9) in (c) prior to bowel cleansing (prior) and after bowel cleansing (post) using metrics stated in (a). Prior to bowel cleansing, samples are labelled orange, while samples collected post-bowel cleansing (t = 0 hours) are labelled in blue. Microbial clustering of luminal content and biopsy samples based on Bray-Curtis dissimilarities was determined in (d). Biopsy samples are labelled in a red circle and luminal content is labelled in a green triangle in the PCoA plot. Microbial clustering for luminal content samples in (e) and biopsy samples in (f) are depicted with a PCoA plot. Non-parametric Mann-Whitney U-test for alpha diversity and Adonis test for beta diversity were used to identify the statistically significant difference between groups and *p* < 0.05 is considered significant.

To extend these observations, we assessed all five participants and compared microbial diversity between unprepared (unpurged) samples and post-cleansing (purged) samples collected at 0, 8, 12, and 24 hours. Alpha diversity (Observed ASVs, Shannon, Simpson) showed no significant differences between luminal and biopsy samples at baseline (Figure 3a). Post-cleansing luminal samples displayed slightly higher Shannon and Simpson indices, whereas biopsy samples exhibited marginally higher Observed ASVs but lower Shannon and Simpson values (Figure 3b and c). These inconsistent shifts and substantial inter-individual variability indicate that bowel cleansing does not systematically alter alpha diversity in either compartment (*p* > 0.05 for all comparisons), likely reflecting subject-specific responses and heterogeneous purging effects across both luminal and mucosal microbial niches.

In contrast, beta diversity analysis demonstrated compartment-specific sensitivity to purging. Luminal samples showed no significant compositional differences between pre- and post-cleansing states (*p* = 0.285; Figure 3e). However, biopsy samples exhibited a significant post-cleansing shift (*p* < 0.05; Figure 3f), suggesting that the mucosa-associated microbiota is more vulnerable to bowel preparation than the luminal community. Baseline luminal and biopsy samples did not differ significantly in their overall composition (*p* = 0.642; Figure 3d), highlighting that the perturbation arises specifically from the purging process rather than intrinsic differences between sample types.

### Variation of bacterial communities between volunteers

Given the absence of consistent shifts in alpha diversity and the compartment-specific effects observed in beta diversity, we next examined the extent to which microbial composition was shaped by inter-individual variation rather than by bowel cleansing or sample type. Human microbial communities are known to be highly personalized,^44^^,^^45^ and our dataset strongly reflected this hallmark. Across all samples, Bray-Curtis dissimilarity revealed clear clustering by participant identity (*p* < 0.001; Supplementary Figure 1a). This pattern was confirmed using both unweighted and weighted UniFrac metrics (Supplementary Figure 1b and 1c), indicating that individuality dominated the microbial landscape irrespective of bowel cleansing or sampling compartment.

Interestingly, weighted UniFrac analysis uncovered a secondary layer of structure: samples from participants P3, P4, and P5 grouped together (Cluster 1), whereas samples from P1, P2, and P6 formed a distinct cluster (Cluster 2). Because this separation emerged only when relative abundance was considered—disappearing under the presence/absence-only unweighted UniFrac, it likely reflects subtle, abundance-driven differences in a subset of taxa. Nevertheless, PERMDISP analysis showed comparable dispersion among individuals (*p* > 0.05), confirming that the observed clustering resulted from genuine subject-specific profiles rather than heterogeneity in variance.

Altogether, these findings underscore that inter-individual variation is the dominant driver of microbial community structure in healthy adults. Any effects of bowel cleansing must therefore be interpreted within the context of strong baseline microbial “fingerprints”, which can mask or outweigh short-term perturbations introduced by purging.

### Short-term effect of bowel cleansing on the mucosa-associated microbiota ecosystem

**Figure 4.**
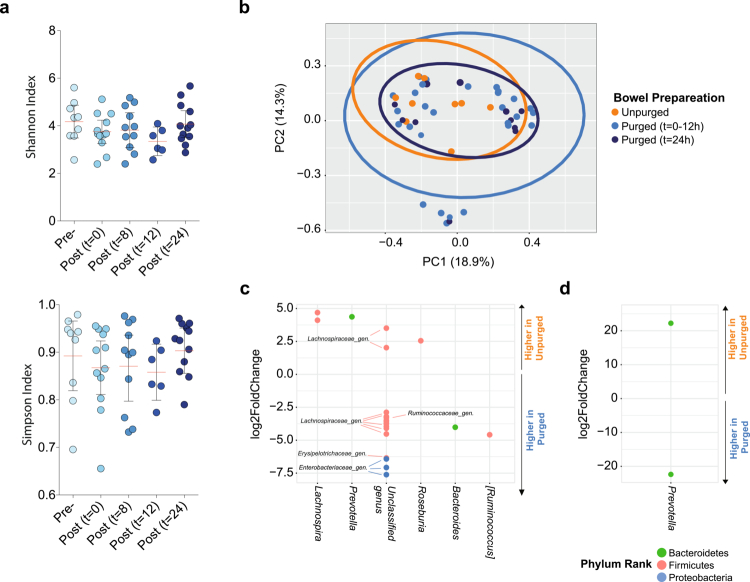
Rapid restoration of cleansing-induced microbial perturbations in biopsies. (a) Shannon and Simpson indices across time points (Pre-, 0-24 h post-cleansing). (b) Bray-Curtis PCoA showing compositional shifts immediately after cleansing and progressive return toward baseline by 24 h (note that nearly all samples at 24 h cluster with unpurged profiles). (c) DESeq2 analysis identifying significantly altered taxa in the first 12 h post-cleansing. (d) DESeq2 analysis showing taxa significantly altered at 24 h. *p* < 0.05 and q < 0.05 were considered significant for (a-b) and (c-d), respectively. Mucosal biopsy and luminal sample numbers are: pre-cleansing *n* = 8; 0 h post-cleansing *n* = 12; 8 h *n* = 11; 12 h *n* = 6; 24 h *n* = 12.

Because individuality strongly dominated overall microbial variation, we next focused on within-subject dynamics to determine how bowel cleansing perturbs the mucosa-associated community over time. In biopsy samples, alpha diversity (Shannon and Simpson indices) showed a transient decrease following bowel cleansing, with the most pronounced reductions occurring within the first 12 hours (*p* > 0.05; Figure 4a). Although these changes did not reach statistical significance, the pattern suggested an immediate disturbance to mucosal species richness that largely resolved by 24 hours, at which point diversity metrics returned to levels comparable to the pre-cleansing state.

Beta diversity analysis provided stronger evidence of disruption. Bray-Curtis dissimilarity revealed a significant shift in community composition immediately after bowel preparation (*p* < 0.01; Figure 4b). Importantly, biopsy samples collected 24 hours post-cleansing clustered closely with pre-cleansing samples, indicating substantial recovery of the mucosa-associated microbiota within this period. This temporal structure was reflected taxonomically: the families *Bacteroidaceae, Prevotellaceae, Erysipelotrichaceae, Lachnospiraceae, Ruminococcaceae,* and *Enterobacteriaceae* showed the most notable fluctuations during first 12 hours after purging.

Differential abundance analysis at 12 hours post-cleansing identified six ASVs that were significantly more abundant prior to bowel cleansing and eighteen ASVs that were significantly increased after cleansing (Figure 4c). ASVs enriched in pre-cleansing biopsies included members of *Prevotella, Coprococcus, Lachnospira, Roseburia,* as well as unclassified taxa from the *Ruminococcaceae* and *Lachnospiraceae* families. In contrast, post-cleansing biopsy samples exhibited higher abundances of ASVs belonging to *Ruminococcus* and *Bacteroides*, as well as taxa from *Erysipelotrichaceae, Enterobacteriaceae, Lachnospiraceae,* and *Ruminococcaceae.*

By 24 hours post-cleansing, these taxonomic differences had largely disappeared. Only two ASVs belonging to the *Prevotella* genus remained significantly altered relative to baseline (Figure 4d), consistent with the observed convergence of beta diversity back to the pre-cleansing state (Figure 4b). Analysis of samples stratified by time point confirmed that the strongest perturbation was detectable at colonoscopy (t = 0h) and during the 8-12 h window after purging, after which the mucosal community reassembled toward its original configuration.

In contrast to biopsies, luminal content samples showed minimal compositional disruption. Neither alpha diversity (Supplementary Figure 2a) nor beta diversity (Supplementary Figure 2b) differed significantly between pre- and post-cleansing states. Notably, luminal samples collected immediately after purging were nearly indistinguishable from their pre-cleansing counterparts, and this stability effectively functions as an internal negative control. The absence of perturbation at the luminal level indicates that the sequencing workflow and downstream analyses do not generate spurious variation, thereby validating that the compositional shifts observed in mucosal biopsies represent genuine biological effects of bowel preparation rather than methodological artefacts. Consistent with this, DESeq2 analysis detected only a mild but significant increase in two ASVs belonging to the Enterobacteriaceae family in luminal samples collected after bowel cleansing (Supplementary Figure 2c), underscoring the minimal impact of purging on this compartment.

Together, these findings show that bowel cleansing induces a rapid but largely reversible restructuring of the mucosa-associated microbial ecosystem, driven by transient changes in specific taxa within the Clostridiales, Bacteroidales, and Enterobacteriales orders.

### Taxonomic changes in rectal microbiota upon bowel cleansing are being restored to the initial microbiota

**Figure 5.**
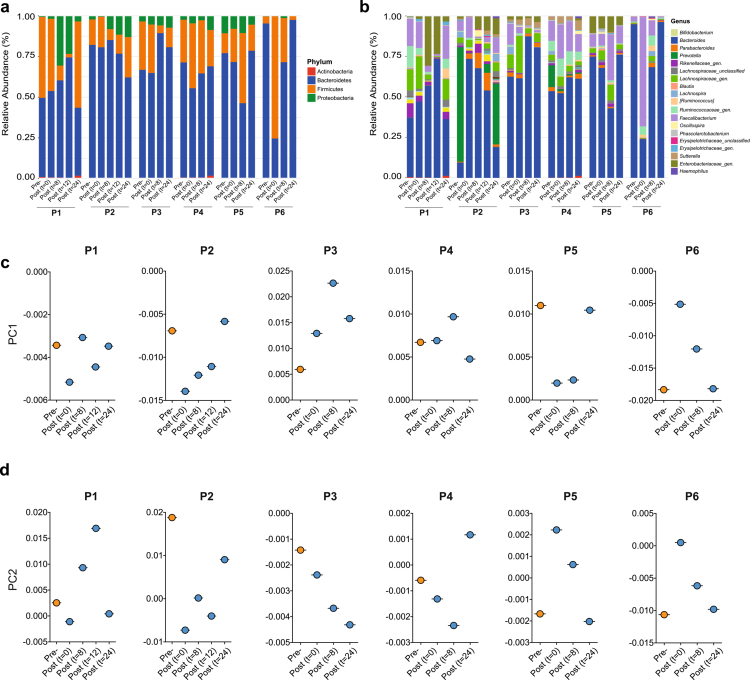
Taxonomic profiles before and after bowel cleansing. Taxonomy profile in rectal mucosal biopsies of the subject at the phylum level (a) and the genus level (b) is shown with different time points post-bowel cleansing. The volunteers are labelled as P1 - P6. Microbial changes within 24 hours post-bowel cleansing for each individual. Principal coordinates analysis (PCoA) of Bray-Curtis distances represented with the first two components in (c) for PC1 axis and in (d) for PC2 axis. Each point represents a single time point for each subject (P1-P6) labelled at the top of each graph. Please note exemplary in PC1 the full return to basal level in P6, P5, P2 and P1. Likewise, as for PC2, the same can be appreciated at least for P6, P5, and P1. As for P3 and P4, a clear trend to baseline levels can be seen, but most likely would have needed some more time. The “*unclassified” term* is used to identify an unclassified genus, while the “*gen.” term* is for an unnamed genus.

To characterize the short-term taxonomic dynamics induced by bowel cleansing, we compared microbial profiles across all time points at both the phylum and genus levels (Figure 5). At the phylum level, relative abundance patterns showed marked inter-individual variation, with subject-specific shifts involving Firmicutes, Bacteroidetes, and Proteobacteria (Figure 5a). These changes were heterogeneous across participants: for example, the Bacteroidetes-to-Firmicutes ratio increased in subjects P3 and P6 but decreased in others (Supplementary Figure 3a). Despite this variability, one consistent pattern emerged: biopsy samples displayed a transient rise in Proteobacteria, predominantly immediately after purging. This increase was detectable in all participants except P5 and P6, and although not statistically significant (*p* > 0.05), it aligns with prior observations in both healthy and diseased cohorts.^22-24^^,^^26^^,^^27^^,^^46^

To improve interpretability, it is important to note that the principal coordinate shifts observed in Figure 5 are modest in amplitude. This pattern is consistent with previous reports showing that bowel cleansing induces only small, transient alterations in mucosa-associated communities, and reflects the strong, individualized baseline structure that constrains the magnitude of within-host changes even when statistically significant. Accordingly, the small but reproducible displacements in PCoA space represent true biological perturbations rather than technical variation.

By 24 hours post-cleansing, the relative abundance of Bacteroidetes in most subjects had returned to pre-cleansing levels, whereas Firmicutes remained slightly reduced. This likely reflected the temporary expansion of Proteobacteria during early recovery. To resolve these dynamics at higher taxonomic resolution, we performed multivariable association analysis using MaAsLin2, incorporating age and sex as covariates (Figure 5b; Supplementary Figure 3b). While most genus-level changes were modest and did not reach significance (*p* > 0.05), several reproducible trends emerged. Across participants, we observed post-cleansing increases in *Bacteroides, Parabacteroides, Sutterella, Haemophilus, [Ruminococcus]*, and unclassified Enterobacteriaceae taxa. Conversely, *Prevotella,* unclassified members of *Rikenellaceae* and *Ruminococcaceae*, and *Lachnospira* showed relative decreases or remained stable.

To visualize community-level recovery, we examined the principal coordinate axes that captured the greatest variance in community structure (Figure 5c and d). Immediately after purging, samples from all participants exhibited clear displacement from their baseline positions. By 24 hours, however, most samples had returned toward pre-cleansing coordinates on both PC1 and PC2. Subjects P6, P5, P2, and P1 recovered nearly completely, while P3 and P4 showed strong trajectories toward baseline but may have required slightly more time to reach full restoration. These temporal trajectories provide an integrated view of microbiota resilience, demonstrating that while bowel cleansing induces compartment-specific and individualized taxonomic fluctuations, particularly involving members of *Bacteroidaceae, Prevotellaceae, Lachnospiraceae, Ruminococcaceae, Erysipelotrichaceae,* and *Enterobacteriaceae*, the microbial communities largely re-establish their original configuration within 24 hours.

While the analyses above establish the magnitude, timing, and individuality of mucosa-associated perturbations after bowel cleansing, they do not resolve which mucosal strata drive these effects. We therefore next applied our mucus-harvesting protocol to directly interrogate spatially distinct mucus-associated compartments.

### Mucus-resolved sampling reveals discrete microbial niches at the rectal mucosal interface

While our longitudinal analysis demonstrated that mucosa-associated microbiota undergo more pronounced compositional shifts following bowel cleansing than luminal communities, the spatial micro-architecture of these mucosal niches remains insufficiently explored in vivo. The outer mucus layer, in particular, constitutes a dynamic biophysical interface between luminal microbes and the intestinal epithelium, yet this layer has rarely been directly sampled in humans. To systematically characterize the microbial consortia inhabiting this interface, we developed an endoscopy-based mucus aspiration protocol that selectively harvests the outer mucus layer from the rectum.

**Figure 6.**
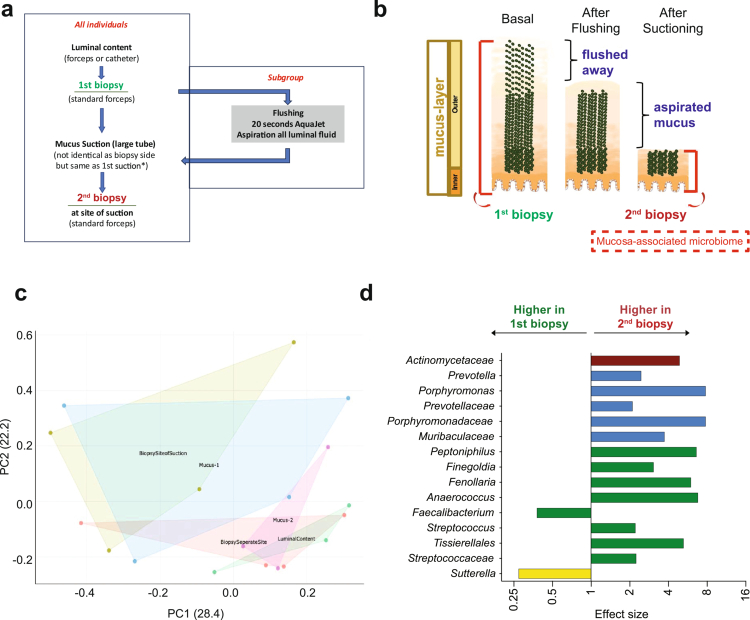
Compartmental analysis using mucus-harvest protocols. (a) Minimum harvest protocol for each subject: luminal content collection, baseline biopsy at one site, mucus aspiration (via suction using a large-bore catheter), and a second biopsy at the same site to capture the mucosa-associated community. Subgroups additionally underwent: (i) vigorous 20-second aqua-jet flushing prior to aspiration, and (ii) sequential mucus aspiration twice at the same site. (b) Schematic of the working model of compartments resolved by the harvesting protocol. The mucus layer (shades of orange) includes an outer layer and a deeper inner layer (the latter presumed sterile in healthy individuals). Green symbols mark taxa enriched in Biopsy 2, representing mucosa-associated microbes (see also panel d). From left to right, the schematic illustrates each procedural step: a baseline biopsy, flushing that removes the superficial outer mucus, aspiration of residual outer mucus, and a second biopsy capturing deeper mucosa-associated communities. (c) Bray-Curtis PCoA for one individual showing differences between biopsies, mucus aspirates, and sequential aspiration manoeuvres. (d) Differentially abundant taxa between Biopsy 1 (baseline) and Biopsy 2 (post-aspiration) across unpurged conditions (*n* = 21), highlighting microbes enriched in the mucosa-associated compartment.

The mucus gel of the colon is known to form a distinct ecological niche enriched for bacterial taxa that thrive in glycan-rich, low-shear environments. Consistent with prior experimental studies,^10^ mucus aspirates obtained through our protocol revealed a microbial community composition clearly distinguishable from luminal aspirates and standard biopsies. The sampling workflow, comprising luminal aspiration, targeted mucus suction, a pre-suction biopsy, and a second biopsy after mechanical mucus removal; enabled spatial deconvolution of microbial communities across these compartments (Figure 6a). Applying the full protocol to one individual examined twice several months apart demonstrated robust reproducibility, with each compartment maintaining a characteristic taxonomic signature across procedures (Figure 6a−c). To directly assess the technical reproducibility of the mucus-harvesting procedure, we additionally compared paired mucus samples collected from the same individuals on two separate days using the identical aspiration protocol. These paired samples exhibited low Bray-Curtis dissimilarity and highly concordant genus-level profiles, indicating consistent recovery of mucus-associated microbial communities across sampling days (Supplementary Figure 4). Importantly, the second biopsy obtained after mucus aspiration and flushing (Biopsy 2) consistently differed from the initial biopsy collected under native conditions (Biopsy 1), indicating that removal of the superficial mucus layer exposes a deeper mucosa-associated microbial community that is distinct from the overlying mucus-resident compartment (*p* < 0.05).

Differential abundance analyses reinforced this compartmentalization. Several genera, *including Sutterella, Streptococcus, Tissierellales, Faecalibacterium, Peptoniphilus, Finegoldia,* and unclassified members of *Porphyromonadaceae*, were significantly enriched in Biopsy 2 relative to Biopsy 1 (Figure 6d). These taxa likely represent the more adherent, mucosa-associated consortia that are shielded beneath the superficial mucus. In contrast, the aspirated mucus samples captured a distinct community enriched for Enterobacteriaceae, suggesting that facultative anaerobes preferentially colonize the outer mucus layer rather than the deeper mucosal surface.

Together, these findings demonstrate successful isolation of spatially distinct microbial niches within the rectum using the mucus aspiration protocol. The ability to sequentially remove mucus layers and compare biopsies before and after mucus displacement provides direct in vivo evidence for stratified microbial organization at the luminal-mucosal interface. This methodological advance opens new opportunities to interrogate microbiota-host interactions in a spatially resolved manner, revealing microbial consortia that are otherwise inaccessible through standard luminal or biopsy-based sampling (Figure 6).

### Enterobacteriaceae enrichment reveals niche-specific sensitivity to bowel cleansing and mechanical disruption.

**Figure 7.**
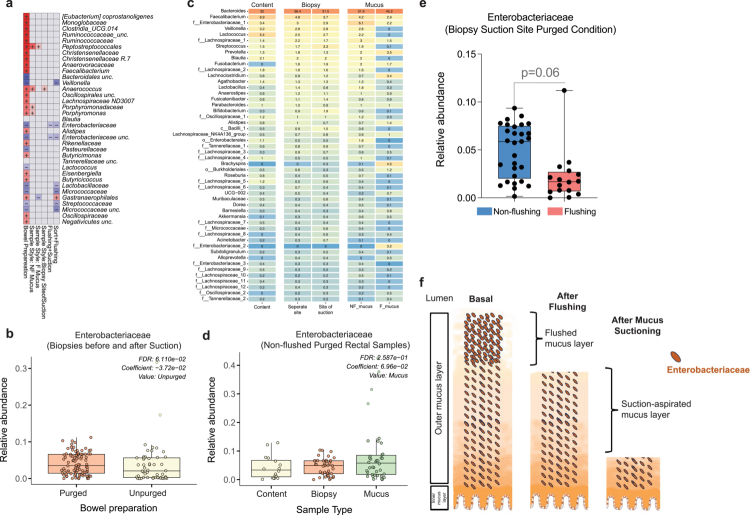
Effects of purging and flushing on Enterobacteriaceae in mucus-associated microbiota. (a) Overview of how bowel preparation, flushing, and mucus aspiration shape gut microbial composition. (b) Effect of bowel cleansing on relative abundance of Enterobacteriaceae. (c) Heatmap comparing bacterial taxa across compartments; superficial mucus shows enriched Enterobacteriaceae, whereas post-flushing mucus is relatively enriched in *Brachyspira, Lachnoclostridium*, and *Prevotella.* (d) Relative abundance of Enterobacteriaceae across purged intestinal content (yellow), biopsies (orange), and mucus aspirates (green), illustrating their preferential localization to the mucus layer. (e) Enterobacteriaceae abundance with or without vigorous flushing. (f) Proposed model of Enterobacteriaceae compartmentalization: enrichment in the superficial outer mucus layer, which is susceptible to removal by aqua-jet flushing, consistent with its loosely structured and oxygen-exposed properties.

To further characterize how bowel cleansing perturbs specific taxonomic groups across intestinal compartments, we focused on *Enterobacteriaceae*, a facultative anaerobic family known to expand under ecological disturbance. Multivariate taxonomic modelling identified several bacterial families whose abundances were significantly altered following purging, with Enterobacteriaceae showing one of the most consistent responses (Figure 7a). Across all subjects, samples collected after bowel cleansing displayed a marked increase in relative abundance of Enterobacteriaceae (Figure 7b), aligning with multiple reports linking Proteobacteria expansion to dysbiosis, rapid transit, or diarrheal states.^47^^,^^48^

Stratified analysis across compartments revealed a striking niche specificity. Aspirated mucus samples exhibited the highest levels of Enterobacteriaceae, markedly exceeding those found in luminal contents or biopsies (Figure 7c and d). This preferential enrichment indicates that the outer mucus layer serves as a microenvironment in which facultative anaerobes have a competitive advantage likely driven by its higher oxygen availability, richer glycoconjugate content, and dynamic structural properties.

Supporting this interpretation, vigorous flushing with an endoscopic aqua-jet markedly reduced *Enterobacteriaceae* abundance at the targeted site (Figure 7e). Mechanical displacement removed the loosely adherent, superficial mucus layer where Enterobacteriaceae were concentrated, revealing a deeper microbial consortium enriched *in Brachyspira, Lachnoclostridium*, and *Prevotella* (Figure 7c). These taxa are characteristic of more stable, anaerobic mucosal habitats and likely reside beneath the perturbation-prone superficial mucus.

Finally, the integrated spatial representation (Figure 7f) highlights the hierarchical organization of the distal colonic niche. Enterobacteriaceae were concentrated within the easily detachable outer mucus layer, whereas the deeper mucus strata contained a more diverse anaerobic community characteristic of a more stable and oxygen-restricted environment. In contrast, the mucosa-accessible biopsy layer displayed yet another distinct compositional profile, reflecting the deeper, tightly adherent microbial consortium that remains after superficial mucus removal. Together, these spatial patterns underscore the multi-layered architecture of the rectal microenvironment and the differential susceptibility of each compartment to perturbation.

This spatial layering directly explains why *Enterobacteriaceae* expansion after bowel cleansing appears almost exclusively in the superficial mucus compartment, rather than uniformly across colonic habitats. Such localization underscores the need for spatially resolved sampling approaches, as traditional luminal or biopsy-based methods overlook perturbations confined to the outer mucus niche.

## Discussion

Multiple human studies have examined how bowel preparation affects the gut microbiome, primarily focusing on mucosa-associated colonic bacteria after intensive purging with laxatives and a clear-liquid diet.^22-24^^,^^26^^,^^27^^,^^46^ However, the temporal dynamics and compartment-specificity of these changes remain incompletely understood. By integrating luminal content, mucosal biopsies, and an in vivo mucus aspirate, our study provides the first high-resolution, short-term trajectory of purging-induced microbial alterations across distinct colonic niches. In addition, we introduce a novel endoscopic mucus-harvesting protocol that enables direct sampling of the superficial mucus layer, a compartment largely inaccessible in prior work.

Using this approach, we identify three central principles: i) microbial disruption induced by bowel cleansing is detected predominantly in mucosal rather than luminal communities; ii) responses are strongly individual-specific; and iii) most alterations resolve rapidly, with microbial profiles returning toward baseline within 24 hours. Notably, the bloom of *Enterobacteriaceae* occurred almost exclusively within the superficial mucus layer and was markedly reduced by targeted flushing, underscoring the need for spatially resolved sampling when assessing mucosal microbiota. Although luminal and biopsy-based analyses provide essential context, the primary insight of this study emerges from resolving mucus-specific microbial compartments that are invisible to conventional endoscopic sampling.

PicoPrep® increases intraluminal water content and accelerates transit, resulting in efficient evacuation of luminal material. While this process substantially reduces microbial biomass,^46^ we observed no significant shifts in alpha or beta diversity in luminal content after cleansing, consistent with prior stool-based studies.^27^^,^^46^ Taxonomic composition in the lumen appeared largely preserved, suggesting that many taxa are washed out proportionally, producing a dilution effect rather than selective reshaping. In contrast, mucosal biopsies displayed a clear, transient dysbiosis-like signature after purging, with significant changes in beta diversity and differential abundance. These compartment-specific effects align with earlier reports demonstrating that biopsy-derived communities (rich in adherent, oxygen-sensitive taxa) are more vulnerable to bowel prep than stool or luminal samples. Our findings therefore reinforce that biopsy-based microbiome studies must interpret post-prep data in the context of this acute perturbation.

Inter-individual variation was a dominant feature of our dataset, overshadowing purging-related effects in cross-sectional comparisons. Consistent with established literature demonstrating strong person-specific microbial signatures in healthy adults,^24^^,^^49-51^ samples clustered robustly by individual across compartments and time points. This individuality underscores the importance of longitudinal within-subject analyses for detecting transient perturbations; subtle shifts induced by bowel cleansing would be largely masked in between-subject comparisons.

Within individuals, mucosal communities underwent measurable but reversible restructuring. Alpha diversity showed a short-lived decline, and beta diversity analysis revealed significant compositional displacement immediately after purging, with most subjects returning to near-baseline structure by 24 hours. Taxonomically, the most consistent early shifts involved blooms in Proteobacteria, particularly *Enterobacteriaceae*, patterns previously associated with dysbiosis, diarrheal states, and shifts in luminal oxygen tension.^22^^,^^25^^,^^46^^,^^52^ These perturbations likely reflect alterations in microenvironmental conditions during and after lavage, including changes in oxygenation, nutrient exposure, and shear forces.

The spatial mapping afforded by our mucus-harvesting protocol revealed an important refinement: the *Enterobacteriaceae* bloom was not uniformly distributed but was localized almost entirely to the superficial mucus layer. This glycan-rich, partially oxygenated, and loosely adherent niche is distinct from both luminal content and deeper mucosa-associated communities. Its ecological properties likely favor facultative anaerobes capable of rapid proliferation. Vigorous flushing removed this outer layer and sharply reduced Enterobacteriaceae abundance, exposing a deeper consortium enriched for *Brachyspira, Lachnoclostridium, Prevotella*, and other anaerobic taxa. These observations align with foundational concepts of mucus stratification, where the outer mucus layer contains more dynamic, disturbance-sensitive communities and the inner layer harbors more stable, adherent microbiota.^9^^,^^10^ By capturing these layers separately, our approach reveals microbial niches that standard biopsies or luminal aspirates cannot resolve.

Several ecological and physiological mechanisms likely explain why facultative anaerobes, particularly Enterobacteriaceae, preferentially expand in the mucus compartment after bowel cleansing. Purging introduces oxygen into the colon and washes out many obligate anaerobes, transiently reducing the community’s oxygen-scavenging capacity and generating a more oxygenated microenvironment that selectively favors facultative anaerobes.^53^ In parallel, bowel cleansing may transiently alter anaerobic microbial communities at the mucosal interface; however, functional consequences such as effects on short-chain fatty acid (SCFA) production remain speculative in the absence of direct metabolomic measurements. Although we did not directly measure SCFA or directly butyrate availability in this study, prior work has shown that reduced butyrate supply can cause colonocytes to shift from *β*-oxidation to glycolysis, increasing epithelial oxygen leakage.^54^ Such changes in epithelial oxygenation would create conditions that favor organisms capable of aerobic or microaerophilic respiration.

*Enterobacteriaceae* are uniquely equipped for such conditions, as they can exploit oxygen-dependent pathways such as formate oxidation, providing a competitive advantage when oxygen becomes available. These bacteria are also intrinsically adapted to the mucus niche. The outer mucus layer contains abundant glycosylated proteins that serve as nutrient sources for *Enterobacteriaceae,*^55^ including E. coli, which can use mucus glycoconjugates as major carbon substrates.^11^ In murine models, successful E. coli recolonization after antibiotic-induced depletion requires the ability to grow specifically within the mucus compartment.^56^ Mucus also provides alternative nutrient classes, such as phosphatidylserine, which can serve as a sole carbon and nitrogen source for *Enterobacteriaceae.*^57^

Together, these established ecological and metabolic features provide a plausible framework for why the superficial mucus layer constitutes a permissive niche for *Enterobacteriaceae* following bowel cleansing. If bowel preparation induces even mild mucosal inflammation, a phenomenon documented in biopsy-based studies^58-61^ and known to exacerbate inflammatory bowel disease flares,^62^ the accompanying inflammatory oxygenation may further amplify Proteobacteria expansion, consistent with their hallmark role in inflammation-driven dysbiosis.^63^ Our mucus-targeted sampling approach thus provides a spatially resolved framework to examine how mechanical and metabolic perturbations during bowel preparation shape host–microbe interactions at the mucosal interface.

The rapid reconstitution of the microbiota within 24 hours highlights the intrinsic resilience of the colonic ecosystem. Despite the mechanical and chemical disruption associated with bowel cleansing, microbial communities returned toward individualized baselines with remarkable consistency. This resilience is consistent with ecological models of microbiome recovery after transient disturbances such as infection, osmotic diarrhea, or dietary restriction. Importantly, the luminal microbiota showed minimal disruption at any time point, reinforcing that the primary site of perturbation, and subsequent recovery, is the mucosal interface rather than the bulk luminal community. The mucus layer likely plays a key role in reseeding, acting as a reservoir for anaerobic taxa that can repopulate the lumen or epithelial surface once normal physiological conditions are restored. Notably, the transient, mucus-specific bloom of Enterobacteriaceae observed immediately after cleansing had fully resolved by 24 hours, consistent with a short-lived ecological disturbance rather than persistent dysbiosis. Compared with the prolonged or incomplete recovery typically observed after antibiotics, infectious diarrhea, or severe dietary shifts, the rapid restoration seen here suggests that the mucus environment supports a tightly constrained and highly resilient recolonization process. This deeper mucus reservoir is biophysically protected, enriched in anaerobic microzones, and insulated from the mechanical shear and oxygen influx associated with purging, likely enabling it to serve as the principal source of reseeding during recovery.

Our study also refines methodological considerations for human microbiome research. Prior studies differed in laxative regimens, timing of sampling, or reliance on stool rather than mucosal material, which may explain conflicting reports on the extent of disruption. Standard colon-preparation effects measured in stool do not necessarily reflect mucosal changes, and biopsy-based studies that do not account for superficial mucus removal may inadvertently sample mixed layers. By delineating luminal, mucus-associated, and mucosal compartments, our approach provides a clearer framework for interpreting microbiome data obtained during clinical endoscopy.

Several limitations merit consideration. Although direct histological or microscopic validation of mucus layer depth was not feasible in humans due to ethical and technical constraints inherent to endoscopic sampling, the applied approach is grounded in well-established biophysical principles of mucus stratification that have been rigorously validated in animal models.^64^^,^^65^ Multiple internal lines of evidence, including technical reproducibility across days and systematic divergence between mucus aspirates and paired biopsies, support compartment-specific sampling rather than sampling variability.

Second, cohort size was modest; however, the longitudinal within-subject design provides robust internal comparisons and reduces inter-individual variability, a dominant feature of the human gut microbiome. Third, the use of 16S rRNA sequencing constrains fine-scale taxonomic inference and limits functional interpretation. Although this approach was chosen to ensure consistency across low-biomass samples, particularly mucosal biopsies with high host DNA content, deeper metagenomic or meta-transcriptomic profiling would further refine strain-level resolution and functional potential.

A related limitation is the absence of direct functional readouts, such as metabolomic profiling. While our spatially resolved sampling strategy delineates compartment-specific and time-dependent taxonomic dynamics with high resolution, integrating metabolomics or other functional assays in future studies will be essential to directly link microbial restructuring to metabolic activity and host-microbe interactions during recovery from bowel cleansing. Finally, although the mucus aspiration protocol effectively captures the superficial outer mucus layer, accessing the inner mucus layer remains technically challenging in humans without disrupting epithelial integrity. Despite these limitations, the combined temporal and spatial framework presented here provides a unique and mechanistically informative view of microbiota responses to acute perturbation at the human mucosal interface.

In summary, this study defines the short-term impact of bowel preparation on luminal, mucus-associated, and mucosa-associated bacterial communities, revealing rapid but compartment-specific perturbations followed by near-complete recovery within 24 hours. By introducing a novel endoscopic mucus-harvesting method, we uncover microbial strata that have been largely overlooked in human studies, with important implications for understanding host-microbe interactions at the epithelial interface. These findings provide a methodological and conceptual framework for future research on spatially resolved dynamics of the microbiota in health and disease.

## Supplementary Material

Supplementary Material SUPPLEMENTARY FIGURE LEGENDS

## Data Availability

The raw and processed data that support the findings of this study are openly available in the *Figshare* repository (doi: 10.6084/m9.figshare.30630701).

## Supplemental material

Supplemental data for this article can be accessed at https://doi.org/10.1080/19490976.2026.2635866 and https://doi.org/10.6084/m9.figshare.30630701.

## References

[1] Tamboli CP, Neut C, Desreumaux P, Colombel JF. Dysbiosis in inflammatory bowel disease. Gut. 2004;53:1–4. doi: 10.1136/gut.53.1.1. 14684564 PMC1773911

[2] Kaur N, Chen CC, Luther J, Kao JY. Intestinal dysbiosis in inflammatory bowel disease. Gut Microbes. 2011;2:211–216. doi: 10.4161/gmic.2.4.17863. 21983063

[3] Kamada N, Seo SU, Chen GY, Nunez G. Role of the gut microbiota in immunity and inflammatory disease. Nat Rev Immunol. 2013;13:321–335. doi: 10.1038/nri3430. 23618829

[4] Turnbaugh PJ, Ley RE, Mahowald MA, Magrini V, Mardis ER, Gordon JI. An obesity-associated gut microbiome with increased capacity for energy harvest. Natur. 2006;444:1027–1031. doi: 10.1038/nature05414. 17183312

[5] Hsiao EY, McBride SW, Hsien S, Sharon G, Hyde ER, McCue T, Codelli JA, Chow J, Reisman SE, Petrosino JF, et al. Microbiota modulate behavioral and physiological abnormalities associated with neurodevelopmental disorders. Cell. 2013;155:1451–1463. doi: 10.1016/j.cell.2013.11.024. 24315484 PMC3897394

[6] Kamada N, Chen GY, Inohara N, Nunez G. Control of pathogens and pathobionts by the gut microbiota. Nat Immunol. 2013;14:685–690. doi: 10.1038/ni.2608. 23778796 PMC4083503

[7] Lee JWJ, Plichta DR, Asher S, Delsignore M, Jeong T, McGoldrick J, Staller K, Khalili H, Xavier RJ, Chung DC. Association of distinct microbial signatures with premalignant colorectal adenomas. Cell Host Microbe. 2023;31:827–838 e3. doi: 10.1016/j.chom.2023.04.007. 37130517 PMC10477964

[8] La Vecchia M, Clavenna MG, Sculco M, Sala G, Marradi D, Barberis E, Joseph S, Mellai M, Pagano N, Boldorini R, et al. Gut microbiota and metabolome signatures in obese and normal-weight patients with colorectal tumors. iSci. 2025;28:112221. doi: 10.1016/j.isci.2025.112221. PMC11995084 40230532

[9] Gustafsson JK, Hansson GC. Immune regulation of goblet cell and mucus functions in health and disease. Annu Rev Immunol. 2025;43:169–189. doi: 10.1146/annurev-immunol-101721-065224. 39752567

[10] Li H, Limenitakis JP, Fuhrer T, Geuking MB, Lawson MA, Wyss M, Brugiroux S, Keller I, Macpherson JA, Rupp S, et al. The outer mucus layer hosts a distinct intestinal microbial niche. Nat Commun. 2015;6:8292. doi: 10.1038/ncomms9292. 26392213 PMC4595636

[11] Chang DE, Smalley DJ, Tucker DL, Leatham MP, Norris WE, Stevenson SJ, Anderson AB, Grissom JE, Laux DC, Cohen PS, et al. Carbon nutrition of escherichia coli in the mouse intestine. Proc Natl Acad Sci U S A. 2004;101:7427–7432. doi: 10.1073/pnas.0307888101. 15123798 PMC409935

[12] Nava GM, Stappenbeck TS. Diversity of the autochthonous colonic microbiota. Gut Microbes. 2011;2:99–104. doi: 10.4161/gmic.2.2.15416. 21694499 PMC3225773

[13] Mottawea W, Butcher J, Li J, Abujamel T, Manoogian J, Mack D, Stintzi A. The mucosal-luminal interface: an ideal sample to study the mucosa-associated microbiota and the intestinal microbial biogeography. Pediatr Res. 2019;85:895–903. doi: 10.1038/s41390-019-0326-7. 30758325

[14] Juge N. Relationship between mucosa-associated gut microbiota and human diseases. Biochem Soc Trans. 2022;50:1225–1236. doi: 10.1042/BST20201201. 36214382 PMC9704521

[15] Li X, LeBlanc J, Truong A, Vuthoori R, Chen SS, Lustgarten JL, Roth B, Allard J, Ippoliti A, Presley LL, et al. A metaproteomic approach to study human-microbial ecosystems at the mucosal luminal interface. PLoS One. 2011;6:e26542. doi: 10.1371/journal.pone.0026542. 22132074 PMC3221670

[16] Jervis-Bardy J, Leong LE, Marri S, Smith RJ, Choo JM, Smith-Vaughan HC, Nosworthy E, Morris PS, O’Leary S, Rogers GB, et al. Deriving accurate microbiota profiles from human samples with low bacterial content through post-sequencing processing of illumina MiSeq data. Microbiome. 2015;3:19. doi: 10.1186/s40168-015-0083-8. 25969736 PMC4428251

[17] Nagata N, Tohya M, Fukuda S, Suda W, Nishijima S, Takeuchi F, Ohsugi M, Tsujimoto T, Nakamura T, Shimomura A, et al. Effects of bowel preparation on the human gut microbiome and metabolome. Sci Rep. 2019;9:4042. doi: 10.1038/s41598-019-40182-9. 30858400 PMC6411954

[18] Powles STR, Gallagher KI, Chong LWL, Alexander JL, Mullish BH, Hicks LC, McDonald JAK, Marchesi JR, Williams HRT, Orchard TR. Effects of bowel preparation on intestinal bacterial associated urine and faecal metabolites and the associated faecal microbiome. BMC Gastroenterol. 2022;22:240. doi: 10.1186/s12876-022-02301-1. 35562657 PMC9101932

[19] Shaw AG, Black N, Rushd A, Sim K, Randell P, Kroll JS, Epstein J. Assessing the colonic microbiota in children: effects of sample site and bowel preparation. J Pediatr Gastroenterol Nutr. 2017;64:230–237. doi: 10.1097/MPG.0000000000001233. 27070657

[20] Li M, Qian W, Yu L, Tian F, Zhang H, Chen W, Xue Y, Zhai Q. Multi-time-point fecal sampling in human and mouse reveals the formation of new homeostasis in gut microbiota after bowel cleansing. Microorganisms. 2022;10:2317. doi: 10.3390/microorganisms10122317. 36557570 PMC9783159

[21] Shobar RM, Velineni S, Keshavarzian A, Swanson G, DeMeo MT, Melson JE, Losurdo J, Engen PA, Sun Y, Koenig L, et al. The effects of bowel preparation on microbiota-related metrics differ in health and in inflammatory bowel disease and for the mucosal and luminal microbiota compartments. Clin Transl Gastroenterol. 2016;7:e143. doi: 10.1038/ctg.2015.54. 26866392 PMC4817412

[22] Gorkiewicz G, Thallinger GG, Trajanoski S, Lackner S, Stocker G, Hinterleitner T, Gülly C, Högenauer C, Ravel J. Alterations in the colonic microbiota in response to osmotic diarrhea. PLoS One. 2013;8:e55817. doi: 10.1371/journal.pone.0055817. 23409050 PMC3568139

[23] Harrell L, Wang Y, Antonopoulos D, Young V, Lichtenstein L, Huang Y, Hanauer S, Chang E, Singh SR. Standard colonic lavage alters the natural state of mucosal-associated microbiota in the human colon. PLoS One. 2012;7:e32545. doi: 10.1371/journal.pone.0032545. 22389708 PMC3289660

[24] Shobar RM, Velineni S, Keshavarzian A, Swanson G, DeMeo MT, Melson JE, Losurdo J, Engen PA, Sun Y, Koenig L, et al. The effects of bowel preparation on microbiota-related metrics differ in health and in inflammatory bowel disease and for the mucosal and luminal microbiota compartments. Clin Transl Gastroenterol. 2016;7:e143. doi: 10.1038/ctg.2015.54. 26866392 PMC4817412

[25] Drago L, Toscano M, De Grandi R, Casini V, Pace F. Persisting changes of intestinal microbiota after bowel lavage and colonoscopy. Eur J Gastroenterol Hepatol. 2016;28:532–537. doi: 10.1097/MEG.0000000000000581. 27015015

[26] Mai V, Greenwald B, Morris JG, Jr., Raufman JP, Stine OC. Effect of bowel preparation and colonoscopy on post-procedure intestinal microbiota composition. Gut. 2006;55:1822–1823. doi: 10.1136/gut.2006.108266. 17124165 PMC1856456

[27] O'Brien CL, Allison GE, Grimpen F, Pavli P. Impact of colonoscopy bowel preparation on intestinal microbiota. PLoS One. 2013;8:e62815. doi: 10.1371/journal.pone.0062815. 23650530 PMC3641102

[28] Kiesslich R, Schubert S, Mross M, Klugmann T, Klemt-Kropp M, Behnken I, Bonnaud G, Keulen E, Groenen M, Blaker M, et al. Efficacy and safety of PICOPREP tailored dosing compared with PICOPREP day-before dosing for colon cleansing: a multi-centric randomised study. Endosc Int Open. 2017;5:E282–E290. doi: 10.1055/s-0043-102433. 28393103 PMC5382934

[29] Gevers D, Kugathasan S, Denson LA, Vazquez-Baeza Y, Van Treuren W, Ren B, et al. The treatment-naive microbiome in new-onset Crohn's disease. Cell Host Microbe. 2014;15:382–392. 24629344 10.1016/j.chom.2014.02.005 PMC4059512

[30] Sundquist A, Bigdeli S, Jalili R, Druzin ML, Waller S, Pullen KM, El-Sayed YY, Taslimi MM, Batzoglou S, Ronaghi M. Bacterial flora-typing with targeted, chip-based pyrosequencing. BMC Microbiol. 2007;7:108. doi: 10.1186/1471-2180-7-108. 18047683 PMC2244631

[31] Yilmaz B, Juillerat P, Oyas O, Ramon C, Bravo FD, Franc Y, Øyås O, Fournier N, Michetti P, Mueller C, et al. Microbial network disturbances in relapsing refractory Crohn's disease. Nat Med. 2019;25:323–336. doi: 10.1038/s41591-018-0308-z. 30664783

[32] Bolyen E, Rideout JR, Dillon MR, Bokulich NA, Abnet CC, Al-Ghalith GA, Alexander H, Alm EJ, Arumugam M, Asnicar F, et al. Reproducible, interactive, scalable and extensible microbiome data science using QIIME 2. Nat Biotechnol. 2019;37:852–857. doi: 10.1038/s41587-019-0209-9. 31341288 PMC7015180

[33] Schreiner P, Yilmaz B, Rossel JB, Franc Y, Misselwitz B, Scharl M, Zeitz J, Frei P, Greuter T, Vavricka SR, et al. Vegetarian or gluten-free diets in patients with inflammatory bowel disease are associated with lower psychological well-being and a different gut microbiota, but no beneficial effects on the course of the disease. United European Gastroenterol J. 2019;7:767–781. doi: 10.1177/2050640619841249. PMC6620875 31316781

[34] Callahan BJ, McMurdie PJ, Rosen MJ, Han AW, Johnson AJ, Holmes SP. DADA2: high-resolution sample inference from illumina amplicon data. Nat Methods. 2016;13:581–583. doi: 10.1038/nmeth.3869. 27214047 PMC4927377

[35] Pruesse E, Quast C, Knittel K, Fuchs BM, Ludwig W, Peplies J, Glockner FO. SILVA: a comprehensive online resource for quality checked and aligned ribosomal RNA sequence data compatible with ARB. Nucleic Acids Res. 2007;35:7188–7196. doi: 10.1093/nar/gkm864. 17947321 PMC2175337

[36] McMurdie PJ, Holmes S. Phyloseq: an R package for reproducible interactive analysis and graphics of microbiome census data. PLoS One. 2013;8:e61217. doi: 10.1371/journal.pone.0061217. 23630581 PMC3632530

[37] Callahan BJ, Sankaran K, Fukuyama JA, McMurdie PJ, Holmes SP. Bioconductor workflow for microbiome data analysis: from raw reads to community analyses. F1000Res. 2016;5:1492. doi: 10.12688/f1000research.8986.2. 27508062 PMC4955027

[38] Anderson MJ. Distance-based tests for homogeneity of multivariate dispersions. Biometrics. 2006;62:245–253. doi: 10.1111/j.1541-0420.2005.00440.x. 16542252

[39] Morgan XC, Tickle TL, Sokol H, Gevers D, Devaney KL, Ward DV, Reyes JA, Shah SA, LeLeiko N, Snapper SB, et al. Dysfunction of the intestinal microbiome in inflammatory bowel disease and treatment. Genome Biol. 2012;13:R79. doi: 10.1186/gb-2012-13-9-r79. 23013615 PMC3506950

[40] Mallick H, Rahnavard A, McIver LJ, Ma S, Zhang Y, Nguyen LH, Tickle TL, Weingart G, Ren B, Schwager EH, et al. Multivariable association discovery in population-scale meta-omics studies. PLoS Comput Biol. 2021;17:e1009442. doi: 10.1371/journal.pcbi.1009442. 34784344 PMC8714082

[41] McMurdie PJ, Holmes S. Waste not, want not: why rarefying microbiome data is inadmissible. PLoS Comput Biol. 2014;10:e1003531. doi: 10.1371/journal.pcbi.1003531. 24699258 PMC3974642

[42] Love MI, Huber W, Anders S. Moderated estimation of fold change and dispersion for RNA-seq data with DESeq2. Genome Biol. 2014;15:550. doi: 10.1186/s13059-014-0550-8. 25516281 PMC4302049

[43] Edgar RC. Search and clustering orders of magnitude faster than BLAST. Bioinformatics. 2010;26:2460–2461. 20709691 10.1093/bioinformatics/btq461

[44] Human Microbiome Project C Structure, function and diversity of the healthy human microbiome. Natur. 2012;486:207–214. doi: 10.1038/nature11234. PMC3564958 22699609

[45] Rodriguez JM, Murphy K, Stanton C, Ross RP, Kober OI, Juge N, Rodríguez JM, Avershina E, Rudi K, Narbad A, et al. The composition of the gut microbiota throughout life, with an emphasis on early life. Microb Ecol Health Dis. 2015;26:26050. doi: 10.3402/mehd.v26.26050. 25651996 PMC4315782

[46] Jalanka J, Salonen A, Salojarvi J, Ritari J, Immonen O, Marciani L, Salojärvi J, Gowland P, Hoad C, Garsed K, et al. Effects of bowel cleansing on the intestinal microbiota. Gut. 2015;64:1562–1568. doi: 10.1136/gutjnl-2014-307240. 25527456

[47] Lupp C, Robertson ML, Wickham ME, Sekirov I, Champion OL, Gaynor EC, Finlay BB. Host-mediated inflammation disrupts the intestinal microbiota and promotes the overgrowth of enterobacteriaceae. Cell Host Microbe. 2007;2:119–129. doi: 10.1016/j.chom.2007.06.010. 18005726

[48] Zhu W, Winter MG, Byndloss MX, Spiga L, Duerkop BA, Hughes ER, Büttner L, de Lima Romão E, Behrendt CL, Lopez CA, et al. Precision editing of the gut microbiota ameliorates colitis. Natur. 2018;553:208–211. doi: 10.1038/nature25172. PMC5804340 29323293

[49] Chen L, Wang D, Garmaeva S, Kurilshikov A, Vich Vila A, Gacesa R, Sinha T, Segal E, Weersma RK, Wijmenga C, et al. The long-term genetic stability and individual specificity of the human gut microbiome. Cell. 2021;184:2302–2315 e12. doi: 10.1016/j.cell.2021.03.024. 33838112

[50] Lloyd-Price J, Arze C, Ananthakrishnan AN, Schirmer M, Avila-Pacheco J, Poon TW, Andrews E, Ajami NJ, Bonham KS, Brislawn CJ, et al. Multi-omics of the gut microbial ecosystem in inflammatory bowel diseases. Natur. 2019;569:655–662. doi: 10.1038/s41586-019-1237-9. PMC6650278 31142855

[51] Costello EK, Lauber CL, Hamady M, Fierer N, Gordon JI, Knight R. Bacterial community variation in human body habitats across space and time. Sci. 2009;326:1694–1697. doi: 10.1126/science.1177486. PMC3602444 19892944

[52] Demirogullari B, Poyraz A, Cirak MY, Sonmez K, Ozen IO, Kulah C, Karabulut B, Basaklar A, Kale N. Effects of hyperosmolar agents--lactulose, lactitol, sodium phosphate and polyethylene glycol--on cecal coliform bacteria during traditional bowel cleansing: an experimental study in rats. Eur Surg Res. 2004;36:159–164. doi: 10.1159/000077258. 15178905

[53] Byndloss MX, Olsan EE, Rivera-Chávez F, Tiffany CR, Cevallos SA, Lokken KL, Torres TP, Faber F, Gao Y, Litvak Y, et al. Microbiota-activated PPAR-γ signaling inhibits dysbiotic enterobacteriaceae expansion. Sci. 2017;357:570–575. doi: 10.1126/science.aam9949. PMC5642957 28798125

[54] Hughes ER, Winter MG, Duerkop BA, Spiga L, Furtado de Carvalho T, Zhu W, Gillis CC, Büttner L, Smoot MP, Behrendt CL, et al. Microbial respiration and formate oxidation as metabolic signatures of inflammation-associated dysbiosis. Cell Host Microbe. 2017;21:208–219. doi: 10.1016/j.chom.2017.01.005. 28182951 PMC5313043

[55] Sauvaitre T, Van Landuyt J, Durif C, Roussel C, Sivignon A, Chalancon S, Uriot O, Van Herreweghen F, Van de Wiele T, Etienne-Mesmin L, et al. Role of mucus-bacteria interactions in enterotoxigenic escherichia coli (ETEC) H10407 virulence and interplay with human microbiome. NPJ Biofilms Microbiomes. 2022;8:86. doi: 10.1038/s41522-022-00344-6. 36266277 PMC9584927

[56] Wadolkowski EA, Laux DC, Cohen PS. Colonization of the streptomycin-treated mouse large intestine by a human fecal escherichia coli strain: role of growth in mucus. Infect Immun. 1988;56(5):1030–1035. doi: 10.1128/iai.56.5.1030-1035.1988. 3281898 PMC259757

[57] Krivan HC, Franklin DP, Wang W, Laux DC, Cohen PS. Phosphatidylserine found in intestinal mucus serves as a sole source of carbon and nitrogen for salmonellae and escherichia coli. Infect Immun. 1992;60:3943–3946. doi: 10.1128/iai.60.9.3943-3946.1992. 1500206 PMC257417

[58] Rejchrt S, Bures J, Siroký M, Kopácová M, Slezák L, Langr F. A prospective, observational study of colonic mucosal abnormalities associated with orally administered sodium phosphate for colon cleansing before colonoscopy. Gastrointest Endosc. 2004;59:651–654. 15114307 10.1016/s0016-5107(04)00158-0

[59] Mehershahi S, Ghazanfar H, Ashraf S, Shaikh DH, Patel H. Colitis induced by colon-cleansing agent. Case Rep Gastroenterol. 2021;15:621–625. doi: 10.1159/000514440. 34616266 PMC8454223

[60] Lawrance IC, Willert RP, Murray K. Bowel cleansing for colonoscopy: prospective randomized assessment of efficacy and of induced mucosal abnormality with three preparation agents. Endoscopy. 2011;43:412–418. doi: 10.1055/s-0030-1256193. 21547879

[61] Erdogan B, Isiksoy S, Dundar E, Pasaoglu O, Bal C. The effects of sodium phosphate and polyethylene glycol-electrolyte bowel preparation solutions on 2, 4, 6-trinitrobenzenesulfonic acid-induced colitis in the rat. Exp Toxicol Pathol. 2003;55:213–220. doi: 10.1078/0940-2993-00318. 14620544

[62] Menees S, Higgins P, Korsnes S, Elta G. Does colonoscopy cause increased ulcerative colitis symptoms? Inflamm Bowel Dis. 2007;13:12–18. doi: 10.1002/ibd.20049. 17206634

[63] Shin NR, Whon TW, Bae JW. Proteobacteria: microbial signature of dysbiosis in gut microbiota. Trends Biotechnol. 2015;33:496–503. 26210164 10.1016/j.tibtech.2015.06.011

[64] Johansson ME, Phillipson M, Petersson J, Velcich A, Holm L, Hansson GC. The inner of the two Muc2 mucin-dependent mucus layers in colon is devoid of bacteria. Proc Natl Acad Sci U S A. 2008;105:15064–15069. doi: 10.1073/pnas.0803124105. 18806221 PMC2567493

[65] Johansson ME, Larsson JM, Hansson GC. The two mucus layers of colon are organized by the MUC2 mucin, whereas the outer layer is a legislator of host-microbial interactions. Proc Natl Acad Sci U S A. 2011;108(Suppl 1):4659–4665. doi: 10.1073/pnas.1006451107. 20615996 PMC3063600

