# Supplementary material for: Spatial mapping of human colonic niches reveals rapid, mucus-specific microbiota disruption after bowel cleansing
Source: Gut Microbes. 2026 Feb 25;18(1):2635866. doi: 10.1080/19490976.2026.2635866 (PMC12940144; doi:10.1080/19490976.2026.2635866)
Supplement: Supplementary Material — SUPPLEMENTARY FIGURE LEGENDS [file KGMI_A_2635866_SM8576.docx]

**SUPPLEMENTARY FIGURE LEGENDS**

**
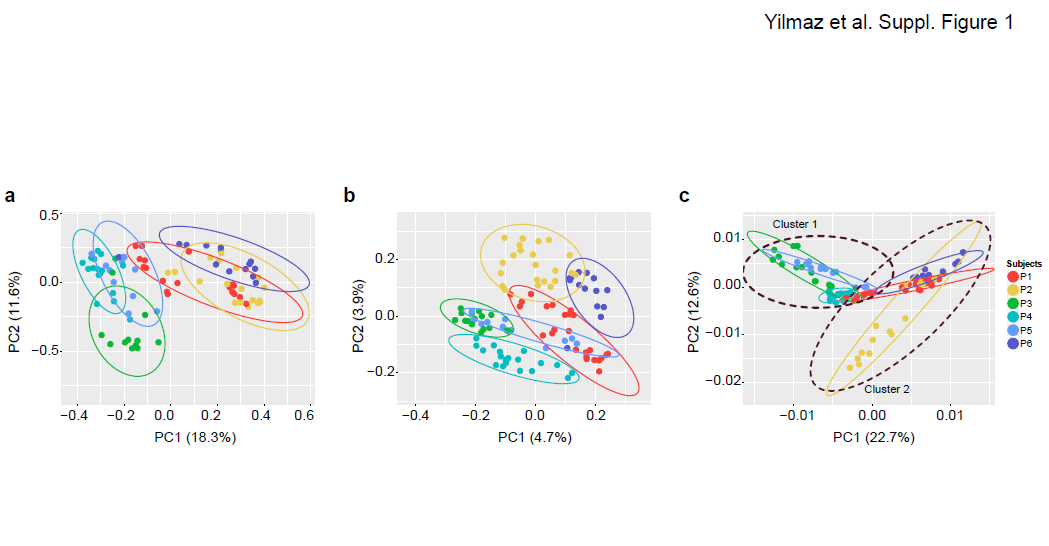
**

**Supplementary Figure 1. Beta diversity analysis for each individual subject.** Beta diversity profiles calculated using Bray-Curtis dissimilarity (a), unweighted UniFrac (b), and weighted UniFrac (c) illustrate subject-specific clustering patterns and highlight the strong inter-individual differences in community structure. Number of samples obtained per participant: P1 (n = 19), P2 (n = 18), P3 (n = 12), P4 (n = 16), P5 (n = 10), and P6 (n = 13). Sample numbers reflect all available rectal and sigmoid biopsies and luminal samples collected before and after bowel cleansing; variation in sample numbers is due to occasional failure to amplify or to pass quality control following 16S rRNA gene sequencing.


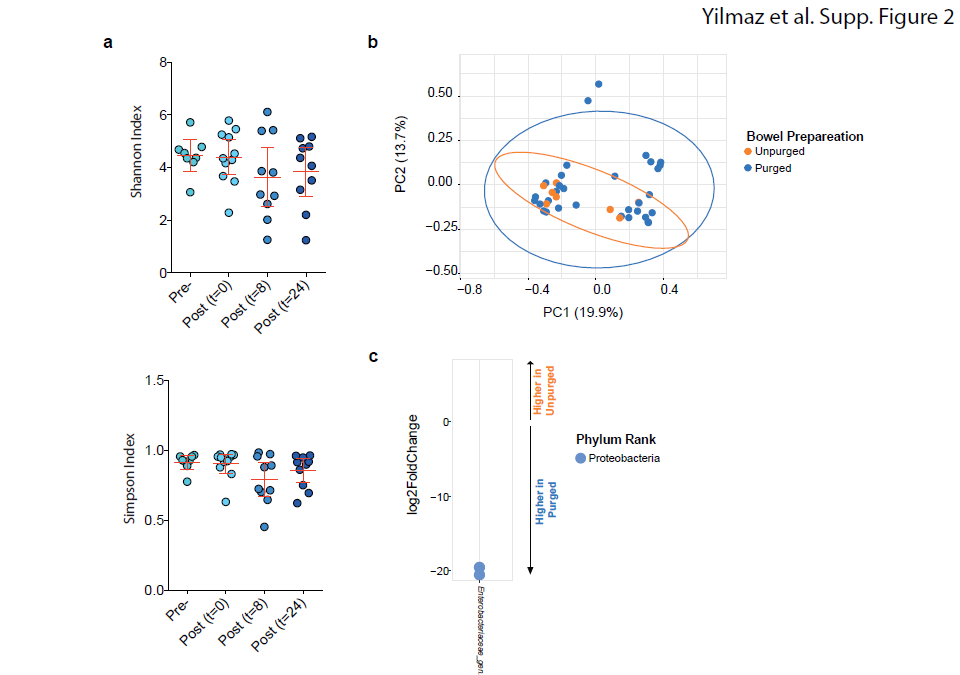


**Supplementary Figure 2. Stable microbiota over time and bowel cleansing in human luminal content samples.** Stability of luminal microbiota over time and after bowel cleansing. (a) Alpha diversity metrics (Shannon and Simpson indices) and (b) beta diversity based on Bray-Curtis dissimilarities show that luminal content samples collected before bowel cleansing (Pre-) and at 0, 8, and 24 h post-cleansing exhibit no significant alterations in overall diversity. (c) DESeq2-based differential abundance analysis identifies two ASVs belonging to Enterobacteriaceae that increase significantly after bowel cleansing, representing the only detectable taxonomic signal in this compartment. p < 0.05 and adj-p < 0.05 were considered significant for (a-b) and (c), respectively. Rectal luminal sample numbers are: pre-cleansing n = 8; 0 h post-cleansing n = 11; 8 h n = 10; 24 h n = 10.


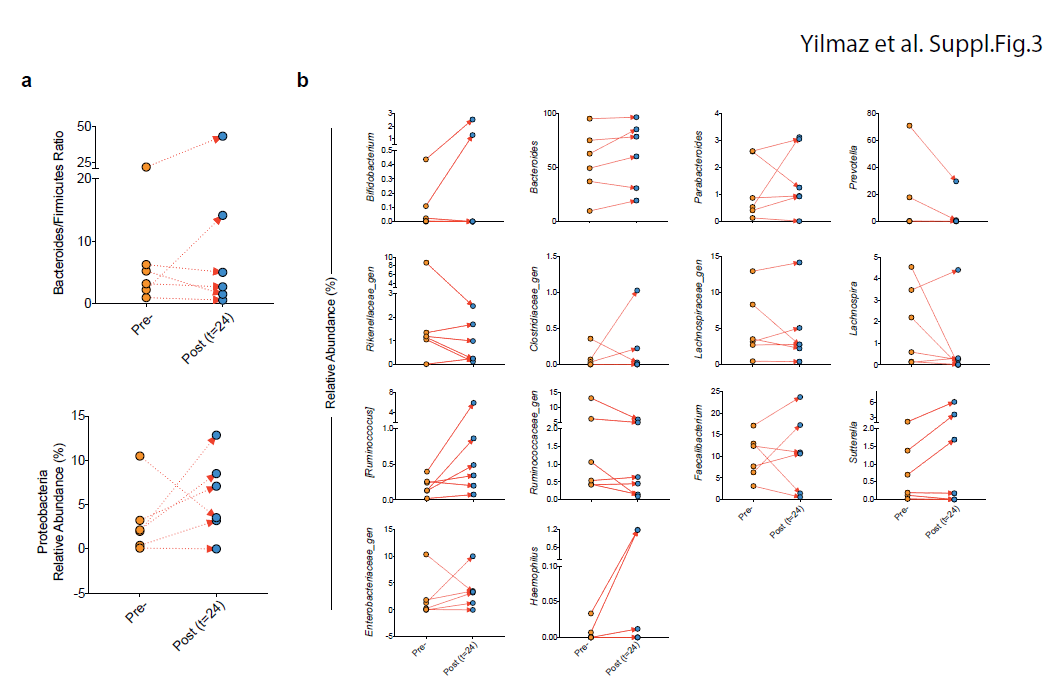


**Supplementary Figure 3. Subject-specific taxonomic changes following bowel cleansing in rectal mucosal biopsies.** (a) Individual trajectories of the Bacteroidetes-to-Firmicutes ratio and Proteobacteria relative abundance demonstrate heterogeneous responses across six subjects. (b) Per-subject changes in key bacterial genera most affected by bowel cleansing, as identified through longitudinal differential abundance analysis. Some taxa are absent or below detection thresholds in certain individuals, therefore the number of comparisons varies between panels.


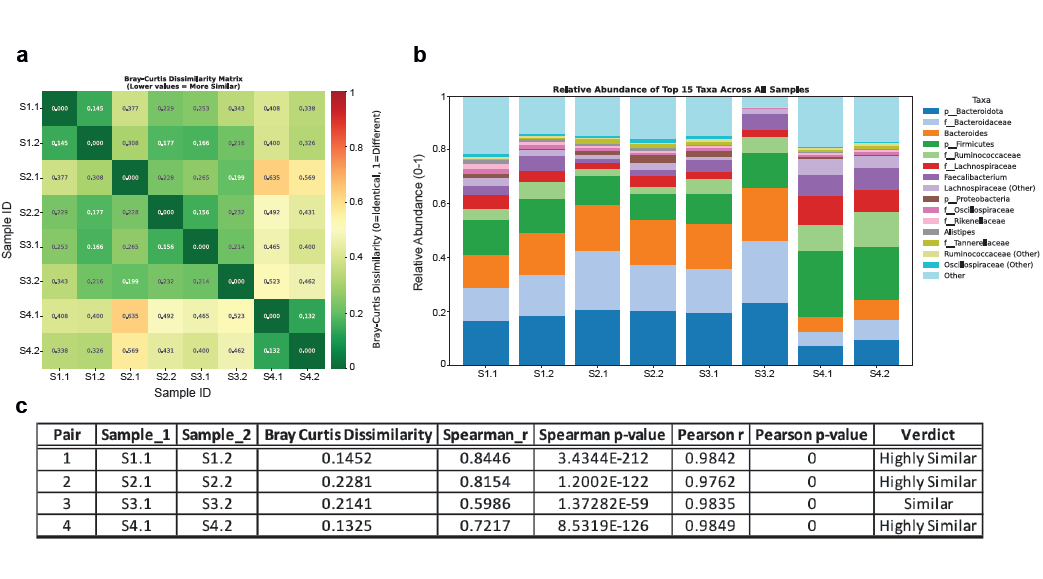


**Supplementary Figure 4**. **Technical reproducibility of endoscopic mucus harvesting.** (a) Bray-Curtis dissimilarity matrix illustrating beta-diversity relationships between sequential mucus samples collected from the same individual on two separate days using the identical aspiration protocol. Low pairwise dissimilarities indicate high compositional similarity between replicate mucus samples. (b) Relative taxonomic composition (Top 15 taxa) of paired mucus samples collected from the same individual using the identical mucus aspiration method, demonstrating highly similar community structures across replicates. (c) Quantitative assessment of reproducibility between paired mucus samples. For each pair, Bray-Curtis dissimilarity and Spearman and Pearson correlation coefficients were calculated based on genus-level relative abundances. All paired samples showed low dissimilarity and high correlation, supporting the technical reproducibility of the mucus-harvesting approach.

**SUPPLEMENTARY MATERIAL**

**Supplementary Video 1. The animation of the mucus harvest protocol**. Under endoscopic visualization, a diagonally bevelled catheter is placed tangentially against the rectal mucosa, and gentle suction is applied with a 50 ml syringe to draw the superficial mucus layer into the tube. A focused aqua-jet rinse is then administered to dislodge and clear this outer mucus compartment. The original aspiration site, usually identifiable by a subtle suction-induced color change, is subsequently shifted, and a post-flush biopsy is collected to enable paired profiling of the deeper mucosa-associated microbiota.
